# Serological Correlates of Protection Induced by COVID-19 Vaccination in the Working Age Population: A Systematic Review and Meta-Analysis

**DOI:** 10.3390/vaccines12050494

**Published:** 2024-05-03

**Authors:** Alborz Rahmani, Alfredo Montecucco, Luca Priano, Lucia Mandolini, Guglielmo Dini, Paolo Durando

**Affiliations:** 1 Department of Health Sciences, University of Genoa, 16132 Genoa, Italy; alfredo.montecucco@edu.unige.it (A.M.); luca.priano@edu.unige.it (L.P.); lucia.mandolini@edu.unige.it (L.M.); guglielmo.dini@unige.it (G.D.); durando@unige.it (P.D.); 2 Occupational Medicine Unit, IRCCS Ospedale Policlinico San Martino, 16132 Genoa, Italy

**Keywords:** surrogate of protection, occupational health, SARS-CoV-2 vaccines, humoral immunity, immune protection

## Abstract

COVID-19 vaccines represent effective public health measures in contrasting the pandemic worldwide. However, protection at the individual-level, which is of crucial importance from an occupational health perspective, is commonly assessed by a serological correlate of protection (CoP) for SARS-CoV-2, which has not yet been determined. The emergence of variants of concern (VOCs) that have shown high rates of breakthrough infections has further complicated the understanding of immune protection against infection. To define a potential serological correlate of protection induced by the COVID-19 vaccination, a systematic review and meta-analysis was performed to summarize the evidence concerning the binding antibody concentration corresponding to a protective effect. Eighteen and four studies were included in the qualitative and quantitative analyses, respectively. The protection against infection was shown for anti-receptor-binding domain (RBD) titers ranging from 154 to 168.2 binding antibody units (BAU)/mL during the pre-Omicron period, while ranging from 1235 to 3035 BAU/mL in the Omicron period. Pooling the results from the studies concerning anti-RBD and anti-Spike antibody titer, we found a mean of 1341.5 BAU/mL and 1400.1 BAU/mL, respectively. These findings suggest that although a fixed serological threshold corresponding to protection against different SARS-CoV-2 variants is not yet definable, higher binding antibody concentrations are associated with increased protective effects.

## 1. Introduction

The rapid clinical development and widespread availability of COVID-19 vaccines since December 2020, a few months after the emergence of the novel SARS-CoV-2, and through extensive implementation of vaccination campaigns at national levels, has been of paramount importance in facing the pandemic worldwide [1]. This significantly contributed to control the global crisis, officially bringing an end to the Public Health Emergency of International Concern (PHEIC) in May 2023 [2]. However, despite the effectiveness demonstrated at the population-level, the existence of a correlate of protection (CoP) for SARS-CoV-2 has not yet been determined. Indeed, adaptive immunity induced by SARS-CoV-2 vaccines is likely both humoral and cellular [3], as already demonstrated for other respiratory infections (e.g., influenza virus), but it is not clear how these two branches can be accurately measured using quantitative diagnostic testing. A CoP is an immunological marker associated with protection from an infectious agent following natural infection or vaccination [4]. It can be distinguished in mechanistic, that is directly responsible for protection, and non-mechanistic or surrogate, which can be used in substitute of the true correlate even though not directly responsible for preventing the infection [4,5]. CoPs can be absolute, where a specific level or threshold of immunological response is highly correlated with protection against infectious disease, or relative, where the level of response is variably correlated with protection, with higher levels of a biomarker associated with more protection [6]. While the lack of standardized, well-validated assays to measure T cell responses has hindered the evaluation of specific T cell responses as a correlate of protection in large-scale settings [7], in the absence of a well-defined humoral CoP, serological testing also cannot be used to confirm immune protection. This has been further complicated by the emergence of variants of concern (VOCs) that have shown high rates of breakthrough infections in previously fully vaccinated subjects [8].

This knowledge gap is particularly impactful in the field of occupational health, where the objective is not only the containment and reduction in infectious risk in the workforce at the population-level, but also the protection of each individual worker exposed to specific biological risks. In this regard, vaccinations represent a fundamental tool to protect at-risk workers that can be recommended by the Occupational Physicians based on the individual workplace, specific professional tasks and health status (e.g., comorbidities, immunocompromised) of each worker.

Therefore, the current systematic review and meta-analysis aims to summarize the available evidence in the literature on the serological CoP induced by SARS-CoV-2 vaccines, with the goal of providing up-to-date and relevant information to occupational physicians and other health professionals, improving both workers’ health assessment as well as the evaluation of SARS-CoV-2 transmission risk in the workplace.

## 2. Materials and Methods

The systematic review and meta-analysis were performed and reported following the Preferred Reporting Items for Systematic Reviews and Meta-analyses (PRISMA) guidelines [9].

A comprehensive search strategy was developed (Appendix A) to gather all research articles reporting correlates/surrogates of protection against SARS-CoV-2 infection induced by the COVID-19 vaccination, in working age individuals between 15 and 64 years, published from 1 January 2020 to 1 December 2023, in English and Italian languages, through systematic searches of three major scientific databases, PubMed/MEDLINE, Scopus and Web of Science. Each source was last searched or consulted on 1 February 2024. Additionally, a manual search of references in the included articles was performed to look for further relevant studies. Study eligibility was defined according to the following PICO criteria: P (population): working age population (in accordance with the Organisation for Economic Cooperation and Development, defined as individuals aged between 15–64) [10], immunized with COVID-19 vaccines; I (intervention): assessment of serological immunity induced by the COVID-19 vaccination; C (comparator): serological immunity induced by different types of COVID-19 vaccines; O (outcome): definition of a humoral correlate of protection induced by vaccination. Case reports, case series, modeling studies, animal studies, environmental sampling studies and review articles, were excluded. When a decision of inclusion or exclusion of a study was not possible to make based on the title and/or abstract, the full text of the study was examined. A comprehensive outlook of inclusion and exclusion criteria is detailed in Appendix A.

Initially, two authors (A.R. and G.D.) independently screened and retrieved eligible articles. At the end of the screening stage, four reviewers (A.R., L.P., L.M., and A.M.) assessed and selected all relevant full-text articles to be included in the systematic review. A Microsoft Excel (version 2402) dataset was created to extract the following variables from each eligible study: name of first author, year of publication, country, study design, sample size, average age, gender ratio, type and proportion of vaccination, proportions of primary immunization cycle and booster doses, proportion of immunocompromised individuals, average time since last dose, proportion of previous SARS-CoV-2 infection, average anti-receptor-binding domain (RBD) IgG and anti-Spike serologic titer corresponding to protection from infection, dominant circulating Variant of Concern (VOC) at the time of study and type of serological assessment assay. Studies were included if the majority of the included sample (50% + 1 threshold) were immunocompetent and did not present immune status suppression/deficiencies. A request of clarification or information was sent to the authors of the studies in case of doubt or lack of data. Quality assessment of included studies was performed independently by two authors (A.R. and G.D.) using the Joanna Briggs Institute Critical Appraisal Checklist tools, for the different study designs included in this review. A third author (A.M.) was involved to resolve disagreements regarding the quality grading.

### 2.1. Data Analysis

For every study included, the mean anti-RBD IgG titer required to protect healthy working age individuals from SARS-CoV-2 infection, with 95% Confidence Intervals (CI) was calculated; when not available, means and Standard Deviations (SD) were estimated using sample size, median and Interquartile Range (IQR) values. To perform this calculation, data were checked for skewness from normality [11], and if they were detected as normal, the estimates were calculated [12,13,14]. In accordance to this methodology [11,12,13,14], the calculations were performed using the online ad-hoc tool available at https://www.math.hkbu.edu.hk/~tongt/papers/median2mean.html (accessed on 24 April 2024). To estimate the pooled effect size, the random-effects model was applied. To graphically represent the studies based on effect size and 95% CI, forest plots were produced. Heterogeneity between studies was evaluated using the I^2^ statistic, with substantial heterogeneity considered when values were higher than 50% [15]. Further stratification was performed relative to study quality to identify sources of variation. Sensitivity analyses by removing individual studies from the meta-analysis were performed in order to assess the robustness of the results. When more than two studies were included in a meta-analysis, potential publication bias was first investigated by visually inspecting the asymmetry of the funnel plot, and if present, by performing the Duval and Tweedie’s trim-and-fill analysis and the Egger’s regression test [16,17]. Meta-regression analysis was performed when a specific variable was present in at least ten studies, to assess the effect of moderators on the pooled effect size. Statistical significance was considered when *p* < 0.05. The Prometa (version 3.0) software was used for all statistical analyses.

### 2.2. Registration and Protocol

This review was not registered. The review protocol is available from the corresponding author on reasonable request.

## 3. Results

**Figure 1:**
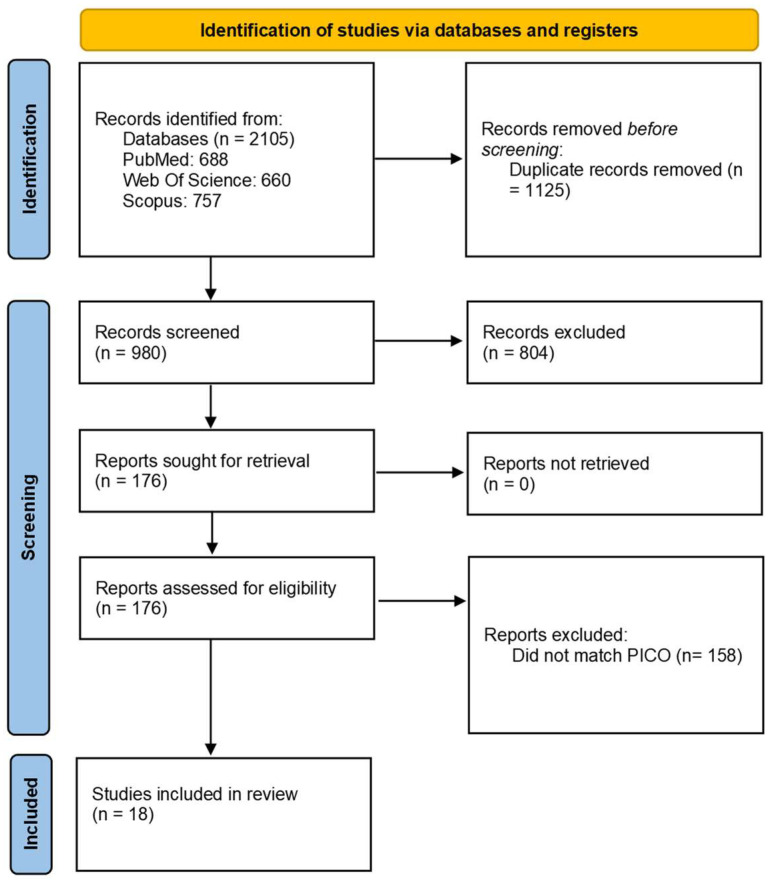
Study selection [9].

The initial systematic search resulted in 2105 potentially relevant articles. After duplicates removal, we obtained a set of 980 unique items. Screening titles and/or abstracts led to excluding 804 items. The remaining articles were sought for retrieval and evaluated in full text. Finally, 18 articles were included in the final analysis after reviewing the eligibility criteria (Figure 1).

Of the included articles, three were performed in Israel and the United States, two in France, Germany, South Africa and Switzerland, while all other countries (Argentina, Brazil, Chile, Colombia, Latvia, Mexico, Peru, Spain, Sweden, United Arab Emirates, United Kingdom) contributed to single studies. With the exception of a single article published in 2021, all articles were published in 2022 and 2023, the majority of which being performed in the latter year (n = 10). Concerning the COVID-19 vaccination type, 16 studies assessed samples obtained from mRNA vaccinated subjects, nine studies from viral vector vaccinated individuals, and one from subjects vaccinated with inactivated vaccines and recombinant protein subunit vaccines. The critical appraisal of the methodological quality of the included studies is reported in Supplementary File S1. The sample sizes ranged from 81 to 222,493 subjects, with a total of 240,431 participants. Between studies, the mean age ranged from 34.9 to 56.0 years; the proportion of female participants ranged from 0.0% to 89.0%; the prevalence of the primary vaccination course completion ranged from 87.4% to 100.0%; and time since last COVID-19 vaccine administration varied from 29.0 to 208.4 days. Finally, most studies assessed vaccine protection during a period where the dominant VOC was Delta and Omicron, with 10 studies assessing each period, while only four studies included samples from pre-Delta VOCs. The main characteristics of the included studies are presented in Table 1.

The qualitative analysis of the included studies showed vastly differing antibody titers corresponding to protective effects against breakthrough infections, particularly when stratifying by the dominant VOC at the time of assessment.

Indeed, while during pre-Delta and Delta pandemic waves, studies showed protection against infection in individuals with anti-RBD titers ranging from 154 binding antibody units (BAU)/mL (95% CI 42–559) [25] to 168.2 BAU/mL [31], and studies performed during the Omicron period showed a protective effect at higher titers, from 1235 [30] to 3035 BAU/mL [8]. During this period, studies showed that antibody levels greater than 2000 BAU/mL (compared to titers lower than 500 BAU/mL) [24] and anti-Spike antibody levels greater than 2816.0 BAU/mL (compared to titers lower than this cut-off) [28] were less likely to become infected, with a 50% reduction in the odds of breakthrough infection. Indeed, studies suggested significant increases in vaccine-induced protection with higher binding antibody concentrations [8,19,23], as was also observed after booster dose administration [28]. Furthermore, Perez-Saez J. et al. suggested that lower levels of anti-S binding antibody, such as 800 units per milliliter, could provide effective protection for Omicron variant in individuals with a history of previous SARS-CoV-2 infection [29].

At the quantitative analysis, data from four studies were obtained [18,20,22,32], as mean antibody titer values corresponding to protective effects against breakthrough infections were reported. Two different meta-analyses were performed in order to pool the findings concerning different types of antibodies.

**Figure 2:**
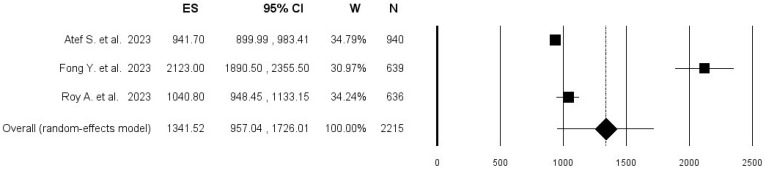
Forest plot of pooled mean anti-RBD antibody titer among subjects who did not acquire breakthrough SARS-CoV-2 infection [18,22,32].

**Figure 3:**
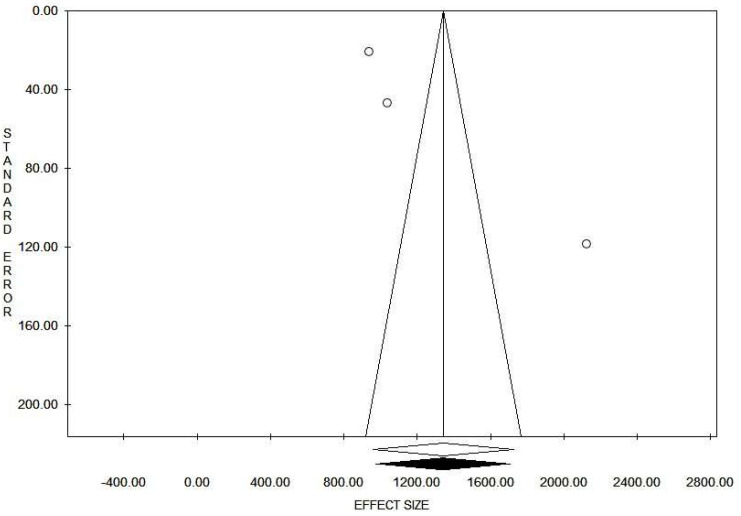
Funnel plot for mean anti-RBD antibody titer among subjects who did not acquire breakthrough SARS-CoV-2 infection.

Concerning the correlate of protection of anti-RBD antibodies, pooling the results from three studies, including 2215 subjects, an overall mean anti-RBD antibody titer among protected individuals of 1341.5 BAU/mL was found (95% CI 957.0–1726.0; I^2^ = 97.9%), with no evidence of publication bias (Figure 2 and Figure 3).

**Figure 4:**
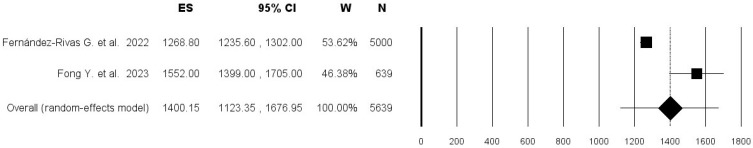
Forest plot of pooled mean anti-Spike antibody titer among subjects who did not acquire breakthrough SARS-CoV-2 infection [20,22].

Pooling the results from the two studies that evaluated anti-Spike antibody titer that demonstrated protection from breakthrough infection, comprising 5639 individuals, an overall mean of 1400.1 BAU/mL was found (95% CI 1123.3–1677.0; I^2^ = 92.0%) in Figure 4.

Meta regression analysis was not performed due to the lack of sufficient number of studies.

## 4. Discussion

To the authors’ knowledge, this systematic review with meta-analysis is the first to specifically study the serological correlate of protection induced by COVID-19 vaccination in the working age population, assessed through readily available and easy to interpret diagnostic assays. A previous systematic review that had investigated the humoral correlate of protection for SARS-CoV-2 suggested that infection could occur in the presence of high levels of antibodies, and thus that a CoP could be relative [35]. Indeed, the findings of the present study suggest that a fixed serological titer corresponding to a protective effect against different SARS-CoV-2 variants is not yet definable. Our study demonstrates that higher binding antibody concentrations are needed to prevent infection from more contagious VOCs. This is particularly evident considering the Omicron variant, with its many subvariants, that has been shown to require much higher binding antibody titers, up to more than 10 times higher compared to previous variants. For this reason, and based on the possible future ecological evolution of the SARS-CoV-2 virus, it is unlikely that a single specific serological cut-off value can be determined to represent protection. Furthermore, it is unclear whether immunization with more recent vaccines, updated with specific antigen sequences/proteins of circulating variants, could provide a more effective protection at lower concentrations. Moreover, although this study assessed the role of specific IgG antibodies, other branches of the immune response could effectively contribute to the protection induced by COVID-19 vaccination, such as IgA, neutralizing antibodies and cellular immunity. In particular, scientific research has focused on the role of mucosal IgA antibodies in the protection from infection, which have been shown to be implicated in the prevention of COVID-19 [26,36], with some authors suggesting that mucosal markers could represent a true mechanistic CoP, while serum titers could be merely non-mechanistic CoP [37]. Regarding cell-mediated immunity, studies have shown that both helper CD4+ T cells and cytotoxic CD8+ T cells perform crucial roles in vaccine-induced protection against COVID-19. Indeed, cytotoxic T cells can recognize and eliminate cells that have been infected by the virus, thus contributing to the control of the viral infection, while helper T cells are essentials for B cells in the development of an effective antibody response. Although cell-mediated immunity can ensure rapid clearance of the virus, possibly reducing the clinical significance and duration of the disease, it may not offer a complete protection from infection. Moreover, the evaluation of virus specific cell-mediated immunity and correlates of protection are more technically complicated and resource-demanding compared to the assessment of the humoral immunity [38].

In light of the above, the evaluation of the different pathways of immune protection are preliminary and require further investigation and validation.

On the other hand, the results of our study suggest that higher anti-RBD or anti-Spike IgG antibody titers could be indicative of a higher protective effect compared to lower concentrations, reinforcing the important role of these antibodies, and their concentrations, in the protection from SARS-CoV-2 infection. This inference is in line with more recently published research, specifically focused on the occupational setting, that investigated a large cohort of healthcare workers in Europe [39]. Moreover, albeit based on a limited number of studies, the meta-analyses in the present investigation could suggest that titers over 1300 BAU/mL could be indicative of a degree of protective effect. Finally, although it was not possible to perform a meta-regression analysis to assess the moderating effect of age on the pooled mean antibody titer, studies that presented a higher mean age in the included sample showed a greater binding antibody concentration corresponding to protection [22,27]. This could be explained by the known association between older age and reduced vaccine efficiency, referred to as immunosenescence, which has previously been demonstrated for different vaccines [40], and that has more recently been suggested also for COVID-19 vaccines [41].

Based on these findings, occupational physicians (OPs) could improve the individual risk assessment and subsequently protect workers exposed to SARS-CoV-2 in the professional setting. Indeed, if these findings are confirmed, the serological assessment of binding antibody concentration could become an additional tool to be included in health surveillance protocols in these occupational settings, possibly aiding in the decision-making process for fitness for work assessments and recommendation for booster vaccination. In this regard it is important to highlight that, in the assessment of risk of infection, as well as of risk of severe disease, on a par with vaccination history, OPs should also take into account prior infections, which contribute significantly to risk reduction in breakthrough infections. Indeed, scientific evidence has shown that hybrid immunity, meaning immunity provided both by natural infection and by vaccination, warrants the highest degree of protection from reinfection and disease severity [42].

The findings of this study are strengthened by the comprehensive and rigorous methodological approach adopted in the literature search and study quality assessment. However, the present investigation presents some limitations, namely the inclusion of few studies, lack of long term follow-up of the protective effect, lack of clear distinction between types and versions of vaccines used in the included studies, lack of effectiveness assessment among immunocompromised individuals, and the evidence of substantial heterogeneity in the quantitative analysis, suggesting ample differences between the included study populations. Indeed, the ample variation in the sample sizes of included studies, as well as the limited number of studies that presented mean protective antibody titers against breakthrough infections reduced the power of the meta-analyses. Specifically, the lack of studies performed during periods with different prevalent circulating variants limited our ability to perform subgroup analyses, which would have been crucial to better assess the effectiveness of COVID-19 vaccines on each VOC. Previous literature has demonstrated that available vaccines show lower estimated effectiveness for the Omicron variant compared to previous ones [43], raising the possibility that a SARS-CoV-2 CoP may be VOC-specific due to evidence showing varying neutralizing ability against different VOCs [44]. Furthermore, the potential effect of waning immunity over time since vaccination could not be adequately assessed as only a few studies followed vaccinees for sufficient time in order to demonstrate effective and prolonged protection from breakthrough infections. Indeed, recent evidence from the literature has shown that this could be a significant factor in the reduction in vaccine-induced protection, potentially requiring periodic booster administration [45]. In this regard, to comprehensively include these time-dependent effects in serology testing and to appropriately identify susceptible, infected, or protected individuals, mathematical models have been developed to take into account both personal and population-level effects [46]. Finally, the comparability of serologic assays, required to assess a true correlate of protection, requires a standardization based on circulating variants. While the present study assessed assays adhering to the WHO international standard, different strategies are being developed to rapidly harmonize assays in light of the evolving variants of SARS-CoV-2 [47]. Studies improving on these limitations could further expand the knowledge and the definition of preventive and protective strategies against SARS-CoV-2.

## 5. Conclusions

In this systematic review and meta-analysis, current evidence regarding the serological correlate of protection induced by COVID-19 vaccination was collected and summarized. In particular, the focus was placed on humoral correlates of protection obtained by means of commonly available serological diagnostic tests, as this could contribute to the rapid translation of research into practice. The present findings show that although it is not yet possible to establish a definite serological correlate of protection induced by the COVID-19 vaccination, higher binding antibody levels demonstrate stronger protective effects among vaccinees.

These results can inform all medical specialties, but can be of particular relevance for occupational physicians, who act as the key players in the prevention and protection of occupational health, especially concerning infectious biological agents. Indeed, they are in a preferential position to assess each individual’s actual exposure to professional risks, based on the workplace, work tasks and specific health susceptibilities, hereby including the presence of serological markers of acquired immunity. Although the primary objective of occupational physicians and other occupational health professionals is to ensure fitness for work and the maintenance and promotion of workers’ health and work capacity, their impact could be consequential also from a public health perspective, as working age individuals represent the majority of the population in developed countries. Further research should be performed to improve knowledge on an absolute correlate of protection, investigating both humoral and cellular immune responses.

## Figures and Tables

**Table 1:** Characteristics of the studies included in the systematic review.

First Name	Year	Country	Study Design	Sample Size (n)	Mean Age (y)	Female (%)	Type of Vaccine	Proportion Primary Course of Vaccination (%)	Proportion First Booster Dose (%)	Proportion Second Booster Dose (%)	Time Since Last Dose (d)	Proportion of Prior Infections (%)	Mean Protective Antibody Titer	Prevalent VOC	Type of Serologic Testing
Atef S. et al. [18]	2023	UAE	Longitudinal	940	35.5	0.0	Inactivated-mRNA	97.7	75.5	2.1	89.2	12.2	Anti-RBD 941.7 (652.4)	Delta	CMIA
Dimeglio C. et al. [19]	2022	France	Longitudinal	259	40.1	74.5	mRNA-VV	100.0	36.7	0.0	208.4	64.9	<6000 BAU/mL provided no protection against Omicron BA.1 infection; 6000–20,000 BAU/mL provided 55.6% protection;20,000 or more provided 87.7% protection	Omicron	ECLIA
Fernández-Rivas G. et al. [20]	2022	Spain	Cross-Sectional	5000	35–54 (44.9)	80.4	mRNA	87.4	NA	NA	180.0	16.6	Anti-Spike 1268.8 (1197.6)	Delta	ECLIA
Fong Y. et al. [21]	2022	Argentina, Brazil, Chile, Colombia, Mexico, Peru, South Africa and USA	Case-cohort	826	49.4	45.2	VV	100	0.0	0.0	29.0	0.0	Breakthrough case 27.54 BAU/mL vs. non-case 32.49 BAU/mL	pre-Delta	ECLIA
Fong Y. et al. [22]	2023	USA	Case-cohort	639	55	46.2	Recombinant protein subunit	100.0	NA	NA	35.0	NA	Anti-RBD 2123.0 (2998.6)- Anti-Spike 1552.0 (1973.3)	pre-Delta	ECLIA
Gilbert P.B. et al. [23]	2022	USA	Case-cohort	1147	54.4	47.0	mRNA	100.0	0.0	0.0	30.0	0.0	spike IgG of 33, 300, and 4000 BAU/mL, vaccine efficacy was 85% (31 to 92%), 90% (77 to 94%), and 94% (91 to 96%)	pre-Delta	ECLIA
Gilboa M. et al. [24]	2023	Israel	Longitudinal	2310	50.0	76.6	mRNA	100.0	100.0	0.0	NA	0.0	IgG > 2000 BAU were less likely to be infected compared to IgG ≤ 500 BAU (OR, 0.52; 95% CI, 0.39–0.67)	Omicron	CMIA
Goldblatt D. et al. [25]	2021	UK–Latvia–South Africa	Cross-Sectional	122	46.5	60.7	mRNA-VV	100.0	NA	NA	15.4	0.0	Overall protective threshold was estimated to be 154 BAU/mL (95% CI 42–559)	pre-Delta. Delta	ECLIA
Hertz T. et al. [26]	2023	Israel	Longitudinal	607	47.3	72.0	mRNA	100.0	100.0	39.9	147.4	0.0	IgG responses against the RBD were not significantly associated with infection status (four doses: *p* = 0.083; three doses *p* = 0.281)	Omicron	ELISA
Macrae K. et al. [27]	2022	Canada	Longitudinal	140	54.6	67.1	mRNA-VV	90.0	56.4	NA	112.3	NA	Average antibody concentration prior to infection was 1911.3 BAU/mL	Delta–Omicron	ELISA
Marking U. et al. [8]	2023	Sweden	Longitudinal	347	52.6	89.0	mRNA-VV	100.0	100.0	0.0	34.4	42.0	Adjusted relative risk of infection for participants above vs. below 75th percentile of serum-IgG was 0.35 (95% CI 0.14–0.71)	Omicron	ECLIA
Möhlendick B. et al. [28]	2022	Germany	Longitudinal	1391	40.7	77.3	mRNA-VV	100.0	100.0	0.0	NA	NA	After 1 month following booster dose administration subjects with 3477.0 BAU/mL became infected, while with 4733.0 BAU/mL did not	Delta–Omicron	CMIA
Perez-Saez J. et al. [29]	2023	Switzerland	Longitudinal	1083	18–64 (91.0)	54.5	mRNA	NA	NA	NA	NA	31.4	Overall three-fold reduction in the hazard of reporting a positive test for antibody levels above 800 IU/mL	Omicron	ECLIA
Regenhardt E. et al. [30]	2023	Germany	Longitudinal	81	34.9	69.1	mRNA-VV	100.0	40.7	0.0	NA	NA	Median anti-RBD-IgG before Omicron breakthrough infection = 1235, 95% CI [771–2404] vs. Delta breakthrough infection = 138, 95% CI [106–220]	Delta–Omicron	CMIA
Regev-Yochay, G. et al. [31]	2023	Israel	Longitudinal	1461	41.7	54.1	mRNA	96.4	0.0	0.0	177.8	22.8	Uninfected 168.2 BAU per mL [95% CI 158.3–178.7] vs. infected 130.5 BAU/mL [118.3–143.8]	Delta	CMIA
Roy A. et al. [32]	2023	France	Longitudinal	636	37.0	74.2	mRNA-VV	100.0	38.1	0.7	120.2	17.1	1040.8 (1188.3)	Delta–Omicron	CMIA
Sendi P. et al. [33]	2023	Switzerland	Longitudinal	949	41.0	27.0	mRNA	89.0	69.5	0.0	NA	54.9	association of anti-S1 IgG levels and protection from infection was higher during the Omicron period	Delta–Omicron	ELISA
Wei J. et al. [34]	2022	UK	Longitudinal	222,493	56.0	53.8	mRNA-VV	100.0	0.0	0.0	71–76	9.7	ChAdOx1 or BNT162b2 required estimated levels of 107 BAU/mL and 94 BAU/mL, respectively	Delta	ELISA

Abbreviations: mRNA—Messenger Ribonucleic Acid Vaccine; VV—Viral Vector Vaccines; CMIA—Chemiluminescent Microparticle Immunoassay; ECLIA—Electrochemiluminescence Immunoassay; ELISA—Enzyme Linked Immunosorbent Assay.

## Data Availability

The data that support the findings of this study are available on request from the corresponding author.

## Supplementary Materials

The following supporting information can be downloaded at: https://www.mdpi.com/article/10.3390/vaccines12050494/s1, Supplementary File S1: Critical appraisal of cohort studies included in the present systematic review; Critical appraisal of cross-sectional studies included in the present systematic review.

## Appendix A. Search strategy

**Search Strategy**	**Details**
**Search query**	(COVID-19 OR SARS-CoV-2) AND (vaccin* OR immunizat* OR vaccinat*) AND (“correlate* of protection” OR CoP OR “surrogate* of protection” OR SoP OR “protective correlate*” OR “protective surrogate*” OR “protective titer” OR “protective titre” OR “vaccine protection” OR “immune protection”)
**Databases**	PubMed/MEDLINE, Scopus, Web of Science
**Time filter**	1 January 2020 to 1 December 2023
**Language filter**	English and Italian
**Inclusion criteria**	P (population): working age population (15–64 years) who underwent COVID-19 vaccination I (intervention): anti-Spike, -S1, -S2, -RBD antibody serologic testing C (comparator): different types of vaccination; different doses of vaccination; immunocompetent vs. immunocompromised O (outcome): mean of anti-Spike and anti-RBD Antibody Titer corresponding to a protective effect, definition of a Correlate of Protection induced by COVID-19 vaccination Study type and design: primary research, studies reporting cross-sectional or longitudinal data
**Exclusion criteria**	Studies not matching the defined PICO criteria; studies on pediatric population; studies on geriatric population; animal studies; reviews; editorials; comments; case-reports; case series
