# Supplementary material for: Serological Correlates of Protection Induced by COVID-19 Vaccination in the Working Age Population: A Systematic Review and Meta-Analysis
Source: Vaccines (Basel). 2024 May 3;12(5):494. doi: 10.3390/vaccines12050494 (PMC11125960; doi:10.3390/vaccines12050494)
Supplement: Supplementary file 1 [file vaccines-12-00494-s001.zip › vaccines-2966182-supplementary.pdf]

Supplementary File S1.

Critical appraisal of cohort studies included in the present systematic review.

**Study**	**Domain 1**	**Domain 2**	**Domain 3**	**Domain 4**	**Domain 5**	**Domain 6**	**Domain 7**	**Domain 8**	**Domain 9**	**Domain 10**	**Domain 11**
Atef S. et al., 2023	Yes	Yes	Yes	Yes	Yes	NA	Yes	No	Yes	NA	Yes
Dimeglio C. et al., 2022	Yes	Yes	Yes	Yes	Unclear	NA	Yes	Yes	Yes	NA	Yes
Fong Y. et al., 2022	Yes	Yes	Yes	Yes	Yes	NA	Yes	No	Yes	NA	Yes
Fong Y. et al., 2023	Yes	Yes	Yes	Yes	Yes	NA	Yes	No	Yes	NA	Yes
Gilbert P.B. et al., 2022	Yes	Yes	Yes	Yes	Yes	NA	Yes	No	Yes	NA	Yes
Gilboa M. et al., 2023	Yes	Yes	Yes	Yes	Yes	NA	Yes	Unclear	Yes	NA	Yes
Hertz T. et al., 2023	Yes	Yes	Yes	Yes	Yes	NA	Yes	Yes	Yes	NA	Yes
Macrae K. et al., 2022	Yes	Yes	Yes	Yes	No	NA	Yes	No	Yes	NA	Yes
Marking U. et al., 2023	Yes	Yes	Yes	Yes	Yes	NA	Yes	No	Yes	NA	Yes
Möhlendick B. et al., 2022	Yes	Yes	Yes	Unclear	No	NA	Yes	Unclear	Yes	NA	Yes
Perez-Saez J. et al., 2023	Yes	Yes	Yes	No	No	NA	Yes	Unclear	Yes	NA	Yes
Regenhardt E. et al., 2023	Yes	Yes	Yes	Yes	Yes	NA	Yes	Unclear	Yes	NA	Yes
Regev-Yochay, G. et al., 2023	Yes	Yes	Yes	Yes	Yes	NA	Yes	Yes	Yes	NA	Yes
Roy A. et al., 2023	Yes	Yes	Yes	Yes	Yes	NA	Yes	Yes	Yes	NA	Yes
Sendi P. et al., 2023	Yes	Yes	Yes	Yes	Yes	NA	Yes	Unclear	Yes	NA	Yes
Wei J. et al., 2022	Yes	Yes	Yes	Yes	Yes	NA	Yes	No	Yes	NA	Yes

Critical appraisal of cross-sectional studies included in the present systematic review.

**Study**	**Domain 1**	**Domain 2**	**Domain 3**	**Domain 4**	**Domain 5**	**Domain 6**	**Domain 7**	**Domain 8**
Goldblatt D. et al., 2021	Unclear	Yes	Yes	Yes	Yes	Yes	Yes	Yes
Fernández-Rivas G. et al., 2022	Yes	Yes	Yes	Yes	Yes	Unclear	Yes	Yes
